# Conformational HIV-1 Envelope on particulate structures: a tool for chemokine coreceptor binding studies

**DOI:** 10.1186/1479-5876-9-S1-S1

**Published:** 2011-01-27

**Authors:** Maria Tagliamonte, Maria Lina Tornesello, Franco M Buonaguro, Luigi Buonaguro

**Affiliations:** 1 Lab. of Molecular Biology and Viral Oncogenesis & AIDS Reference Center, Istituto Nazionale Tumori “Fond. G. Pascale”, Naples, Italy; 2 Institute of Human Virology, University of Maryland School of Medicine, Baltimore, MD, USA

## Abstract

The human immunodeficiency virus type 1 (HIV-1) external envelope glycoprotein gp120 presents conserved binding sites for binding to the primary virus receptor CD4 as well as the major HIV chemokine coreceptors, CCR5 and CXCR4.

Concerted efforts are underway to understand the specific interactions between gp120 and coreceptors as well as their contribution to the subsequent membrane fusion process.

The present review summarizes the current knowledge on this biological aspect, which represents one of the key and essential points of the HIV-host cell interplay and HIV life cycle. The relevance of conformational HIV-1 Envelope proteins presented on Virus-like Particles for appropriate assessment of this molecular interaction, is also discussed.

## Introduction

The molecular interaction between HIV-1 gp120, in its trimeric conformation, and the CD4 receptor on the host cell surface represents the first step of the HIV infection cycle. Upon this interaction, the co-receptor-binding site on the gp120 is exposed, enabling the binding to HIV chemokine coreceptors (mainly CCR5 or CXCR4) expressed on the surface of a subset of CD4+ lymphocytes. The binding to the coreceptors is followed by fusion of the viral and host cell membranes mediated by the HIV gp41 transmembrane glycoprotein [1-6].

Dissecting the structural changes which HIV external envelope glycoprotein gp120 molecule undergo upon molecular interactions with its cognate cellular receptor and coreceptors, provide essential information to the development of HIV-1-specific drugs, targeting the viral entry step [7-16], as well as of vaccines [17-20].

## Gp120 binding to chemokine coreceptors

The HIV-1 Envelope is synthesized as the polyprotein precursor gp160, which undergoes oligomerization, disulfide bond formation and extensive glycosylation in the endoplasmic reticulum [21]. The full post-translational processing and maturation lead to proteolytical cleavage of precursor gp160 into the surface gp120 and transmembrane gp41 subunits by furin-like endo-proteases in the Golgi network [22-24]. The two subunits will assemble into a trimer consisting of three gp120 molecules associated non-covalently with three gp41 subunits.

The molecular interaction of HIV gp120 with the CD4 receptor and, subsequently, with the CCR5 or CXCR4 coreceptor leads to the insertion of the hydrophobic gp41 N-terminal region (fusion peptide) into the host cell membrane. In particular, the gp41 ectodomain trimer acquires the six-helix bundle configuration which drives in close contact the viral and cell membranes, ultimately resulting in their fusion [1,2,4,25,26]. Therefore, the binding of HIV envelope to cellular coreceptors dramatically influence the strength of viral-cell interaction and promote the conformational changes in the gp41 required to overcome the energy barrier and induce pore formation and membrane fusion.

Within the CCR5 and CXCR4 amino acid residues interacting with the gp120, most of the cysteine residues are involved in disulfide bonds formation and play a key functional role. In particular, the N-terminal and second extracellular domain (ECII) of both coreceptors seem to be critical for gp120-CD4 complex binding [27-35].

The role of coreceptors in the conformational changes of the HIV transmembrane gp41 to facilitate virus-cell membrane fusion has not yet been fully clarified, mainly due to the lack of the CCR5 and CXCR4 crystal structure and, therefore, their absence in high resolution X-ray structures solved for CD4-bound gp120 [17]. The currently accepted theory proposes that, upon the coreceptor binding to the gp120-CD4 complex, the gp41 acquires the thermostable, six-helix bundle structure that brings the two membranes together and results in fusion pore formation [36,37].

**Figure 1:**
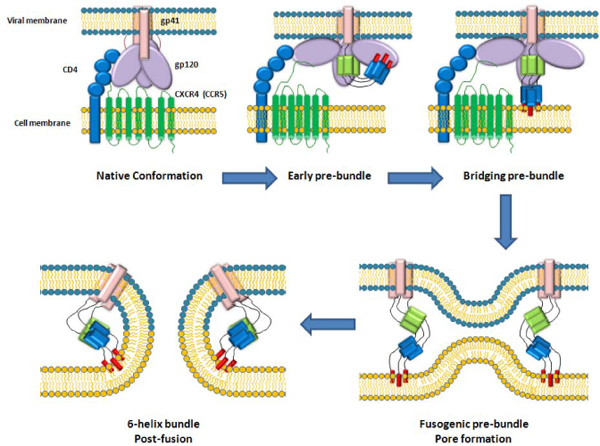
Dissection of sequential steps occurring after engagement of receptor and coreceptor by trimeric HIV envelope proteins.

The first step is the exposure of the hydrophobic fusion peptide at the N terminus of gp41 which interacts with the target cell membrane, generating an intermediate, pre-hairpin state bridging the virus and cell membranes. The pre-hairpin then refolds into the stable, six-helix bundle core structure [38,39], releasing sufficient energy to overcome the kinetic barrier [40,41] and catalyzing the fusion of the two membranes [42]. Whether the fusion can occur with the free energy liberated during refolding of one or several trimers, is still debated [40,43] (Fig.1).

In the described stepwise process, the pre-hairpin state shows a relatively long half-life [44], representing a favorable target for inhibitory peptides [45,46] as well as neutralizing antibodies specific for the gp41 HR1 and MPER regions [47-50].

Several data about the envelope/receptor interactions have been generated also for the simian counterpart of HIV (Simian Immunodeficiency Virus, SIV). Indeed, SIV_mac_ is the natural etiological agent of the AIDS-like syndrome in Rhesus Macaques, which is the only available animal model for obtaining relevant information on AIDS pathogenesis [51-54] as well as for testing efficacy of antiviral therapeutics and vaccine candidates [55,56].

Similarly to HIV-1, SIV infection starts with the high-affinity interaction of the gp120-gp41 envelope glycoprotein (Env) complex with CD4 on the target cell surface [57,58]. However, in contrast to HIV-1, different strains of SIV preferentially use CCR5 and not CXCR4 as coreceptor for entry [59-61], although they may show promiscuity in coreceptor usage, engaging alternative coreceptors GPR15 and CXCR6/STRL33 with high efficiency [62,63].

Moreover, SIV strains have been shown to infect target cells via a CD4-independent pathway, directly interacting with CCR5 or CXCR4 coreceptor [64,65]. It has been proposed that Env protein of such strains contains multiple amino acid substitutions leading to the constitutive exposure of coreceptor binding site [66,67], similarly to what described for CD4-independent HIV-1 envelope proteins [68,69]. As consequence, CD4-independent viruses acquire a broader cell tropism, being able to infect also CD4 negative or low-expressing cells, such as macaque macrophages [70,71].

The molecular interaction of SIV envelope protein with the CD4 receptor and coreceptors leads to conformational changes in the gp41 ectodomain trimers and exposure of the fusion peptide which closely resemble what described for the HIV-1 counterpart [72,73].

### Role of coreceptors post-translational modifications in HIV-1-mediated cell fusion

The effectiveness of CCR5 and CXCR4 as HIV coreceptors depends on the several possible conformations which may significantly influence their ability to support viral entry in different cells [74,75].

Furthermore, post-translational modifications of HIV coreceptors and, in particular, of the extracellular domains (including the N-terminal, EC I – III) and intracellular loops, may modulate the receptor turnover as well as the binding efficacy to the HIV gp120. In general, extracellular domains may undergo N-linked or O-linked glycosylation and tyrosine sulfation, while modifications of intracellular loops include palmitoylation, phosphorylation, and ubiquitination [76-79].

**CCR5.** The CCR5 N-terminal is relevant for the role as HIV-1 coreceptor [27,29,30] and contains several tyrosine residues which may be modified by sulfation, contributing to binding to natural ligands (MIP1-α, MIP1-β) as well as HIV-1 gp120-CD4 complexes [80-82]. In particular, sulfation of Tyrosines at position 10 and 14 seems to be a requisite for the CCR5 binding efficacy [83,84]. Moreover, CCR5 is also modified by O-linked glycosylation, preferentially on Ser-6 [85,86], although it does not seem to affect the role of CCR5 in the HIV entry [86].

As most chemokine receptors, the CCR5 carboxyl-terminus contains one or more cysteine residues compatible with receptor palmitoylation, typically located 12 to 25 amino acids away from the plasma membrane boundary [87]. Cysteine residues at amino acid positions 321, 323, and 324 undergo to palmitoylation, facilitating the CCR5 transport to the plasma membrane as well as ligand-stimulated endocytosis and affecting its ability to initiate intra-cellular signalling pathways [88-90]. However, the biological role of improved localization of CCR5 to lipid rafts for HIV entry into host cells is still disputed [88-92].

**CXCR4.** Similar to CCR5, also CXCR4 undergo post-translational modifications contributing to its function. CXCR4 is sulfated at three tyrosine residues in the N-terminus, with Tyr21 accounting for the majority of sulfate incorporation, although this modification doesn’t appear to modulate the CXCR4 coreceptor function for HIV-1 [83].

In contrast to CCR5, the extracellular domain of CXCR4 is post-translationally modified by N-linked glycosylation in two potential sites, Asn11 and Asn176 [93,94], although only Asn11 appears to be glycosylated in mammalian cells [94]. Mutation of Asn11 does not impair the CXCR4-mediated HIV-1 infection [95,96]; however, a Asn11-to-Glu11 mutation leads to enhanced binding of both CXCR4-specific and dual-tropic (CCR5 and CXCR4) HIV-1 isolates [78,96].

## HIV-1 Envelope-coreceptor signaling

The binding of the gp120-CD4 complex to chemokine coreceptors not only mediates HIV entry but also activates intracellular signaling cascades, mimicking chemokine signaling induced by binding to cognate receptors [97,98] (reviewed in [77,99,100].

However, in addition to signaling pathways mediating cell migration, transcriptional activation, cell growth and differentiation [101-106], binding of gp120-CD4 complex to CCR5 or CXCR4 coreceptor has also been shown to trigger the activation of proline-rich tyrosine kinase (Pyk2), phosphoinositide 3-kinases (PI3K) [107,108] and CD4/CXCR4-dependent NFAT (nuclear factor of activated T cells) nuclear translocation [109]. Furthermore, gp120 was demonstrated to mediate chemotaxis, actin cytoskeleton rearrangement [108], and the activation of an actin depolymerization factor, cofilin, to increase the cortical actin dynamics in resting CD4 T cells [110]. (For a more comprehensive description of gp120-triggered chemokines coreceptor signaling, refer to Cicala and Arthos in this same supplement).

## Stable trimeric forms of human immunodeficiency virus recombinant gp140

As described above, the envelope proteins on the virus surface are assembled into trimers, consisting of three gp120 molecules associated non-covalently with three gp41 subunits, which interact sequentially with the CD4 receptor and the chemokine coreceptors, ultimately leading to viral and cell membrane fusion. In the last years, mainly aiming at inducing more potent and broader anti-HIV neutralizing antibodies, several groups have been developing soluble trimers of the gp120 - gp41 Env ectodomain (i.e., lacking the transmembrane, cytoplasmic domains and named gp140) which are considered to preserve or mimic the structure of functional Env complexes [111-114]. Considering the close structural similarity of these molecules to native trimeric HIV Envelope proteins, this can represent a relevant tool also for receptor and chemokines coreceptor binding studies.

Mutations in the furin cleavage site at the gp120–gp41 junction inhibit the dissociation between the two envelope subunits [114-117], but cleavage-defective gp140 Env proteins seem to be antigenically different from fully processed Env [118-120]. In order to express processed and stable trimers of gp140, an intermolecular disulfide bond has been introduced between the gp120 and gp41 subunits to form a complex called “SOS” gp140 which, although still predominantly monomeric, strongly reacts with the broadly neutralizing b12mAb [118]. An isoleucine-to-proline substitution introduced at position 559 in the N-terminal heptad region of gp41 has been shown to increase the stability of SOS gp140 (SOSIP gp140), leading to a fully cleaved and trimeric structure with optimal antigenic properties [121,122].

Moreover, cleavage-defective gp140 Env proteins have been further modified at the C-terminus to improve trimer formation and stabilization, fusing different heterologous trimerization motifs. In particular, a 32-amino-acid form of the GCN4 transcription factor (GCN), a 27-amino-acid trimerization domain from the C-terminus of bacteriophage T4 fibritin (T4F), or a soluble trimerization domain of chicken cartilage matrix (CART) protein have been employed, showing enhanced binding to broadly neutralizing b12 and 2G12 mAbs [115,116,123]. More recently, the effectiveness of the catalytic chain of aspartate transcarbamoylase (ATCase) as trimerization domain for the HIV gp140 has been described [124,125].

## Exploiting the VLP model

In addition to soluble forms, HIV gp140 trimeric complex can be presented on membrane structures including liposomes, inactivated viruses and virus-like particles (VLPs) or pseudovirions, to mimic as close as possible the native conformation [126-131].

In particular, Virus-like particles (VLPs) represent a complex structure based on viral capsid proteins which self-assemble into particulate structures closely resembling immature virus particles [132-135]. VLPs are replication as well as infection incompetent, lacking regulatory proteins as well as infectious genetic material, and can be considered nanoparticles and employed to deliver antigenic structures as well as DNA molecules to antigen presenting cells, for enhanced induction of immune responses against co-administered plasmid DNA-based immunogens [136-140].

VLPs have been produced from a broad spectrum of non-enveloped and enveloped viruses [140]. In particular, the particle structure of non-enveloped VLPs can be based on single or multiple capsid proteins without a surrounding cell membrane. Examples of such VLPs are those formed by the expression of the major capsid protein of papillomaviruses, parvoviruses and polyomaviruses [135,141-143] or by multiple interacting capsid proteins of Reoviridae family [144]. Alternative strategy for generating non-enveloped virus-like-particles (VLPs) is based on the assembly of the capsid protein derived from RNA bacteriophages [145-147].

Alternatively, enveloped VLPs are based on assembled capsid proteins surrounded by cell membrane and have been developed for enveloped viruses such as hepatitis B and C virus (HBV & HCV), influenza A and retroviruses, including HIV-1 [129,132,133,148-152].

The different forms of VLPs have distinct properties for displaying antigens, given that only enveloped VLPs may display full-length monomeric or multimeric conformational proteins on their surface through trans-membrane domains [129-131,153]. Non-enveloped VLPs, on the contrary, may be employed to present mainly short peptides or protein sequences. This has been achieved either generating chimeric capsid proteins expressing foreign epitope in frame (eg. Gag:V3) [154-157], or chemically linking the foreign epitope to the assembled capsid protein [158], although HIV-1 Gag proteins fused to full-length HIV Reverse Transcriptase (RT) protein, without loosing the capability of assembling into VLPs, have been recently reported [159]. The conformational structure of full-length proteins possibly expressed on non-enveloped VLPs, however, remains to be proven.

As consequence, at present, only enveloped VLPs displaying full-length conformational proteins on their surface may be a suitable experimental model for studies on binding and interaction between HIV envelope and cellular receptor/coreceptors. They, indeed, represent the closest particle structure to native virus.

### Conformational HIV Envelopes presented on particulate structures

Development of HIV-1 Pr55Gag-based VLPs expressing gp120/gp140 trimeric Envelope proteins is a goal pursued by several Groups, using different expression systems (mammalian vs baculovirus) as well as different trans-membrane domains. In particular, our group has previously developed Pr55gag-VLPs presenting a gp120 anchored through the trans-membrane (TM) portion of the Epstein-Barr virus (EBV) gp220/350, showing the formation of oligomeric structures [129] and inducing Env-specific humoral and cellular immune response [160-162]. Additional strategies have been more recently described to express trimeric forms of HIV-1 gp140 molecules on the surface of Pr55gag-VLPs [130,131]. Comparative studies showed that specific trans-membrane domains induced optimal incorporation of gp140 Env trimers onto VLP surface, retaining conserved epitopes and undergoing conformational changes upon CD4 binding [131] (Buonaguro et al., submitted).

**Figure 2:**
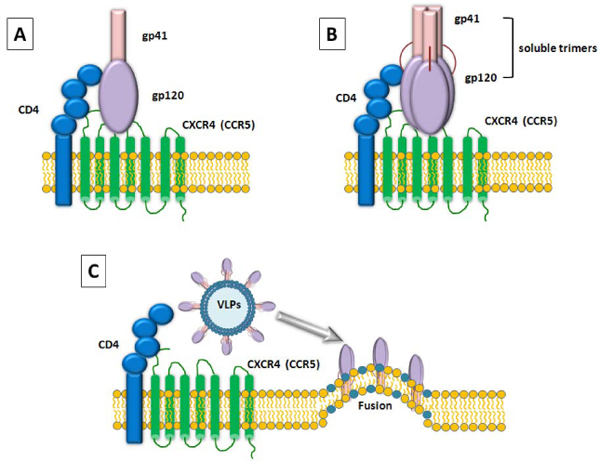
**Schematic representation of HIV-1 gp120 binding to cellular receptor and coreceptors.** The binding of HIV Envelope protein to CD4 receptor and chemokines coreceptors on the host cell surface is represented showing the gp120 in its monomeric form** (A)** and trimeric form as soluble **(B)** or bound to virus like particles (VLPs) **(C).**

Considering the biological and structural properties of HIV gp140 trimers presented on the VLP surface, they can be even more strategic for binding studies, providing an invaluable tool for evaluating and dissecting the whole virus-host cell interaction leading to and ending with membrane fusion [163,164] (Fig. 2).

## Conclusions

The interaction between HIV particles and target host cells is a defined temporally sequential stepwise process, characterized by the binding of surface gp120 Envelope protein to CD4 receptor and subsequent binding of the gp120-CD4 complex to chemokines coreceptors. This will ultimately lead to membrane fusion and host cell infection.

Studies aimed at dissecting the sequential steps of this process have been and will be instrumental not only to fully understand the strategies adopted by HIV to hijack host cells for its own replication but also to develop HIV-1-specific drugs and vaccines.

The development of novel and improved molecular tools, mimicking as close as possible native Envelope trimeric structures expressed on non-infectious particulate structures (i.e. VLPs), will expand the armamentarium for HIV-host cell interaction studies. This will help in shedding further light on such a key moment of the HIV infection as well as pathogenesis.

## Competing interests

The authors declare no competing financial or other interests.
